# A flexible IrO_2_ membrane for pH sensing

**DOI:** 10.1038/s41598-022-15961-6

**Published:** 2022-07-09

**Authors:** Shih-Cheng Chou, Yi-Chieh Hsieh, Wai-Hong Cheang, Bo-Yao Sun, Chao-Yi Chu, San-Yuan Chen, Jung-Chih Chiao, Pu-Wei Wu

**Affiliations:** 1 Department of Materials Science and Engineering, National Yang Ming Chiao Tung University, Hsinchu, 300 Taiwan, ROC; 2 Graduate Institute of Biomedical Science, China Medical University, Taichung, 406 Taiwan, ROC; 3 Department of Electrical and Computer Engineering, Southern Methodist University, Dallas, TX 75205 USA

**Keywords:** Health care, Engineering, Materials science

## Abstract

An optimized mixture of polydopamine (PDA) and polyvinyl alcohol (PVA) is employed as the surface functionalizing agent and reducing agent to encapsulate individual polypropylene (PP) fibers of polypropylene micromembrane (PPMM). The functionalized PPMM becomes hydrophilic to allow the formation of Au nuclei for subsequent electroless Au deposition. The metalized PPMM is further deposited with IrO_2_ nanoparticles, and evaluated as a flexible and porous pH sensor. Images from scanning electron microscope confirms the uniform formation of IrO_2_ nanoparticles on Au-coated PP fibers. For pH-sensing performance, the IrO_2_-decorated metalized PPMM reveals a super-Nernstian response for a sensing slope of -74.45 mV/pH in aqueous solutions with pH value ranging between 2 and 12. In addition, the pH-sensing performance is properly maintained after 5000 bending cycles and hysteresis is modest in an acidic environment. The cell viability test indicates a negligible bio-toxicity. Our strategy of using a conductive polymeric membrane decorated with IrO_2_ nanoparticles enables possible sensing applications in wearable and implantable electronics.

## Introduction

The pH value is a critical indicator for many biological systems^1–3^. It is known that for patients suffering from cancer, renal failure, multiple sclerosis, and psychiatric illness, severe disorders in acid/base equilibrium might occur^4,5^. For example, according to Mani et al., cancer cells preferentially convert glucose and other substances into lactic acid, and hence the intracellular fluid becomes slightly acidic^6–8^. Therefore, an effective pH-sensing technique for tissues is necessary for early diagnosis to prevent further physiological damages. So far, a variety of metal oxides including WO_3_, RuO_2_, and IrO_2_ have been investigated for pH-sensing purpose^9–11^. Among them, the IrO_2_ demonstrates a wide working range, an impressive chemical inertness, and a negligible susceptibility to redox interference.

The IrO_2_ is a conductive oxide known for excellent biocompatibility^12^. In addition to pH-sensing, the IrO_2_ has been explored as a pseudocapacitor for energy storage, as a neuron-stimulating electrode for implantable electronics, and as an oxygen-evolving electrocatalyst in water electrolysis^13–15^. However, the natural reserve for IrO_2_ is limited so its expensive cost prohibits its widespread use in commercial products. Therefore, it is necessary to develop a suitable processing technique to produce IrO_2_ nanoparticles or thin films in a more effective way. Previously, we have deposited IrO_2_ nanoparticles on an ITO substrate, and reported their impressive super-Nernstian pH-sensing behaviors^16^. In addition, we have prepared IrO_2_ thin films on ITO and FTO substrates for bio-stimulating studies^17,18^. However, those substrates are rigid and thus are not applicable in wearable and implantable electronics, for which a flexible platform is necessary.

For pH-sensing in both in-vivo and in-vitro cases, a desirable flexible substrate also requires sufficient porosity to allow intimate contact of analyte. We recognize that a polymer membrane exhibited ideal attributes since its chemical properties (nature of polymer and hydrophilicity) and physical properties (porosity, pore size, permeability, thickness, mechanical strength, etc.) could be tailored-made to meet the specifications of end applications. In addition, many polymer membranes are mature industrial products with reasonable costs. Unfortunately, conventional polymer membranes are electrically insulating, and a metallization pretreatment is required. To date, the metallization of polymer membranes has been conducted through chemical vapor deposition, physical vapor deposition, and electroless deposition^19–21^. It is noted that both physical and chemical vapor depositions entail vacuum chambers with sophisticated equipment, and the conformal deposition of metals within the polymer membrane is rather difficult. In contrast, the electroless route is relatively simple in terms of processing tools, and is more likely to achieve homogeneous coating throughout the entire polymer structure.

In this work, we demonstrated the fabrication of a flexible pH sensor using polypropylene micromembrane (PPMM) as a porous substrate. Our process started with a mixture of polydopamine (PDA) and polyvinyl alcohol (PVA) as the hydrophilic agent to functionalize individual polypropylene (PP) fibers of PPMM. The PDA also served as the reducing agent to prepare nucleation sites for subsequent electroless Au deposition, for which a conformal formation of Au overcoat encapsulating every PP fiber was achieved. The metalized PPMM was further electrodeposited with IrO_2_ nanoparticles, and then evaluated as a flexible pH sensor.

## Experimental section

### Chemicals and reagents

The PPMM (Rone Scientific; catalog number: 201PP-47-045-50; pore size of 450 nm) was in a circular shape with diameter of 47 nm and thickness of 200 µm. Tris(hydroxymethyl)aminomethane hydrochloride (Tris–HCl), sodium l-ascorbate, acetic acid (CH_3_COOH), and boric acid (H_3_BO_3_) were purchased from Sigma-Aldrich. Dopamine hydrochloride (DA-HCl; the monomer for PDA) was purchased from Acros Organic. PVA (MW: 70 kDa) was purchased from Merck. Potassium hydroxide (KOH), sodium hydroxide (NaOH), sodium thiosulfate (Na_2_S_2_O_3_), sodium sulfite (Na_2_SO_3_), and hydrochloric acid (HCl) were purchased from SHOWA. Anhydrous citric acid (C_6_H_8_O_7_) was purchased from J.T. Baker. Phosphoric acid (H_3_PO_4_) was purchased from Honeywell. Hydrogen tetrachloroaurate (III) trihydrate (HAuCl_4_·3H_2_O) and potassium hexachloroiridate (IV) (K_2_IrCl_6_) were purchased from Alfa Aesar. All these chemicals were obtained in analytical grade and were used without further purification.

### Functionalization of PPMM

The functionalization of PPMM was carried out by a conformal deposition of PDA and PVA on individual PP fibers of PPMM. The PPMM was cleaned by ethanol (99.9 vol%) before its use. First, a buffer solution for Tris–HCl was prepared by mixing deionized water and anhydrous ethanol in 7:3 volume ratio. For the codeposition of PDA and PVA, 40 mg DA-HCl (effective DA amount was 32 mg) was dissolved in 20 mL 10 mM Tris–HCl buffer solution, followed by adding different amounts of PVA (64, 128, and 256 mg). At this stage, the pH of the mixture was 6. Subsequently, minute amount of aqueous 1 M KOH solution was added to adjust the pH to 8.5. Next, the PPMM was submerged in the mixture for 20 h at 25 °C. The immersion step was under constant stirring in ambient atmosphere so the dissolved oxygen was able to initiate the oxidation (polymerization) of DA to PDA. Lastly, the sample was retrieved and washed by deionized water. The PPMM after functionalization of PDA and PVA was denoted as the PPMM@PDA/PVA(1/2), PPMM@PDA/PVA(1/4), and PPMM@PDA/PVA(1/8), respectively. The ratio of 1/2, 1/4, and 1/8 represented the weight ratio of PDA/PVA. The purpose was to determine the optimized ratio of PDA/PVA for hydrophilic treatment of PP fibers in PPMM. For control experiments, we also prepared PDA-coated and PVA-coated PPMM by conducting the synthetic process in identical conditions (using 40 mg DA-HCl or 256 mg PVA), and the resulting samples were denoted as the PPMM@PDA and PPMM@PVA, respectively.

### Deposition of Au and IrO_2_ on functionalized PPMM

The functionalized PPMM (PPMM@PDA/PVA(1/8)) underwent a seeding step by submerging the sample in 0.1 M HAuCl_4_ aqueous solution (pH 5.2) at 25 °C for 10 min. In this step, the catechol group of PDA was able to reduce the adsorbed Au^3+^ ions to form Au nuclei for following electroless Au deposition. Next, the sample was immersed in an electroless Au deposition bath containing 8 mL 1 M citrate acid aqueous solution and 9.8 mL deionized water (the pH for the mixture was adjusted to 6.5 by adding 1 M KOH aqueous solution), as well as 0.4 mL 1 M Na_2_SO_3_ aqueous solution (serving as the reducing agent). Next, 1 mL 0.1 M HAuCl_4_ aqueous solution was added under constant stirring, along with the addition of 0.8 mL 1 M Na_2_S_2_O_3_ aqueous solution (serving as the complexing agent). At this stage, the Na_2_SO_3_ reduced the Au^3+^ ions for the formation of Au^+^ ions that were complexed by Na_2_S_2_O_3_. Afterward, the reduction of Au^+^ ions was proceeded by the addition of 0.792 mg sodium l-ascorbate, and the bath temperature was raised to 30 °C. The formation of conformal Au deposit on individual PP fibers lasted for 2 h to fabricate the metalized PPMM, which was denoted as the PPMM@PDA/PVA(1/8)@Au.

The electrodeposition of IrO_2_ nanoparticles on the metalized PPMM started with the preparation of IrO_2_ colloidal suspension according to what was reported by Zhao et al.^22^. The deposition of IrO_2_ nanoparticles was performed in a three-electrode cell using a Pt foil (2 × 2 cm^2^) and a saturated calomel electrode (SCE) as the counter and reference electrodes, respectively. The metalized PPMM (1 × 1 cm^2^), serving as the working electrode, was subjected to a potentiostatic mode at 0.4 V (vs. SCE) for 30 min. The resulting sample was denoted as the PPMM@PDA/PVA(1/8)@Au@IrO_2_.

### Materials characterization and pH-sensing analysis

The morphologies and microstructures for samples including PPMM, PPMM@PDA, and PPMM@PVA, as well as PPMM@PDA/PVA(1/2, 1/4, 1/8), PPMM@PDA/PVA(1/8)@Au, and PPMM@PDA/PVA(1/8)@Au@IrO_2_ were observed by a scanning electron microscope (SEM, JEOL JSM6700F). Measurements for water contact angles were performed by a SURFTENS-Basic (OEG Co.) to determine the hydrophilicity of PPMM, PPMM@PDA, PPMM@PVA, and PPMM@PDA/PVA(1/2, 1/4, 1/8) in size of 1 × 1 cm^2^. X-ray photoelectron spectroscopy (XPS; Thermo Fisher Scientific, ESCALAB XI^+^) was employed to determine the nature of oxygenated functional groups, and their relative amounts for PPMM and PPMM@PDA/PVA(1/8). Bending test was conducted in a custom-made mechanical device by which the PPMM@PDA/PVA(1/8)@Au@IrO_2_ was bended to 60° repeatedly for 5000 cycles.

To determine its pH-sensing responses, the PPMM@PDA/PVA(1/8)@Au@IrO_2_ in size of 1 × 1 cm^2^ was immersed in Britton-Robinson buffer solutions (0.04 M H_3_BO_3_, 0.04 M CH_3_COOH, and 0.04 M H_3_PO_4_) with pH values adjusted to 2, 3, 4, 6, 7, 8, 10, 11, and 12, respectively. The open-circuit potential (OCP) for PPMM@PDA/PVA(1/8)@Au@IrO_2_ in Britton-Robinson buffer solutions with different pH values was recorded for 200 s to obtain its average and standard deviation. Identical experiments were carried out for samples after bending 1000, 3000, and 5000 cycles, respectively. The hysteresis behavior was explored by recording the OCP in Britton-Robinson buffer solutions with pH value adjusted in the order of 2–3–4–6–7–8–10–11–12–11–10–8–7–6–4–3–2. In each measurement, the OCP was recorded for 200 s and the sample was rinsed with deionized water afterwards. All those electrochemical experiments were conducted by a potentiostat (VersaSTAT4) at 25 °C. The SCE and Pt foil (3 × 3 cm^2^) were used as the reference and counter electrodes, respectively.

### Cell viability test

To evaluate any possible bio-toxicity from PPMM@PDA/PVA(1/8)@Au or PPMM@PDA/PVA(1/8)@Au@IrO_2_, a cell viability test was conducted in which L929 cells (mouse lung fibroblasts, ATCC, USA) were cultured within our samples for 1 and 4 days. The cell viability test entailed the CellTiter 96® AQ_ueous_ One Solution Cell Proliferation Assay Kit (Promega Co.) and 3-(4,5-dimethylthiazol-2-yl)-5-(3-carboxymethoxyphenyl)-2-(4-sulfophenyl)-2H-tetrazolium (MTS). The experiments were carried out in three different groups; 1) 48-well plate only (serving as the control group), 2) PPMM@PDA/PVA(1/8)@Au, and 3) PPMM@PDA/PVA(1/8)@Au@IrO_2_. First, the samples in 0.5 × 0.5 cm^2^ were dipped into ethanol and rinsed with phosphate-buffered saline. Afterward, they were dried in an oven at 50 °C for 48 h. Next, the samples were positioned in 48-well plates for cell viability tests. Before the tests, all samples were sterilized under UV illumination (Germicidal lamp GL15, Sankyo Denki) for 24 h at 25 °C. In each group, every individual well was seeded with 400 μL cell suspension which contained L929 cells, Dulbecco’s Modified Eagle Medium (DMEM; Sigma-Aldrich), and 1 vol% antibiotics (Antibiotic–Antimycotic (100X), Gibco™). The effective cell concentration in suspension was 1 × 10^4^/mL. Subsequently, the 48-well plates were maintained in an incubator (5% CO_2_, 37 °C) for 1 and 4 days to allow cell cultivation. Then, the entire cell suspension was emptied and the 48-well plates were rinsed with phosphate-buffered saline (PBS) three times. Afterward, the 48-well plates were filled with a solution (20 vol% MTS and 80 vol% DMEM), followed by incubation for 1 h at 37 °C to produce formazan crystals. The formazan crystal was formed by the reaction between the MTS and remaining living cells so its amount was proportional to the number of living cells. In addition, the amount of formazan crystal was quantitatively determined by its characteristic absorption peak at 450 nm. Next, the solution was collected for absorbance (A) measurements at 450 nm (Epoch™ 2, BioTek Instruments Inc.) and the cell viability was determined by following formula.

1 $${\text{Cell}}\;{\text{viability}} = {\text{A}}_{\exp } /{\text{A}}_{{{\text{control}}}} \times 100\%$$

where the A_exp_ is the absorbance for our sample, and the A_control_ is the absorbance for the control group.

## Results and discussion

### Material characterization

**Figure 1:**
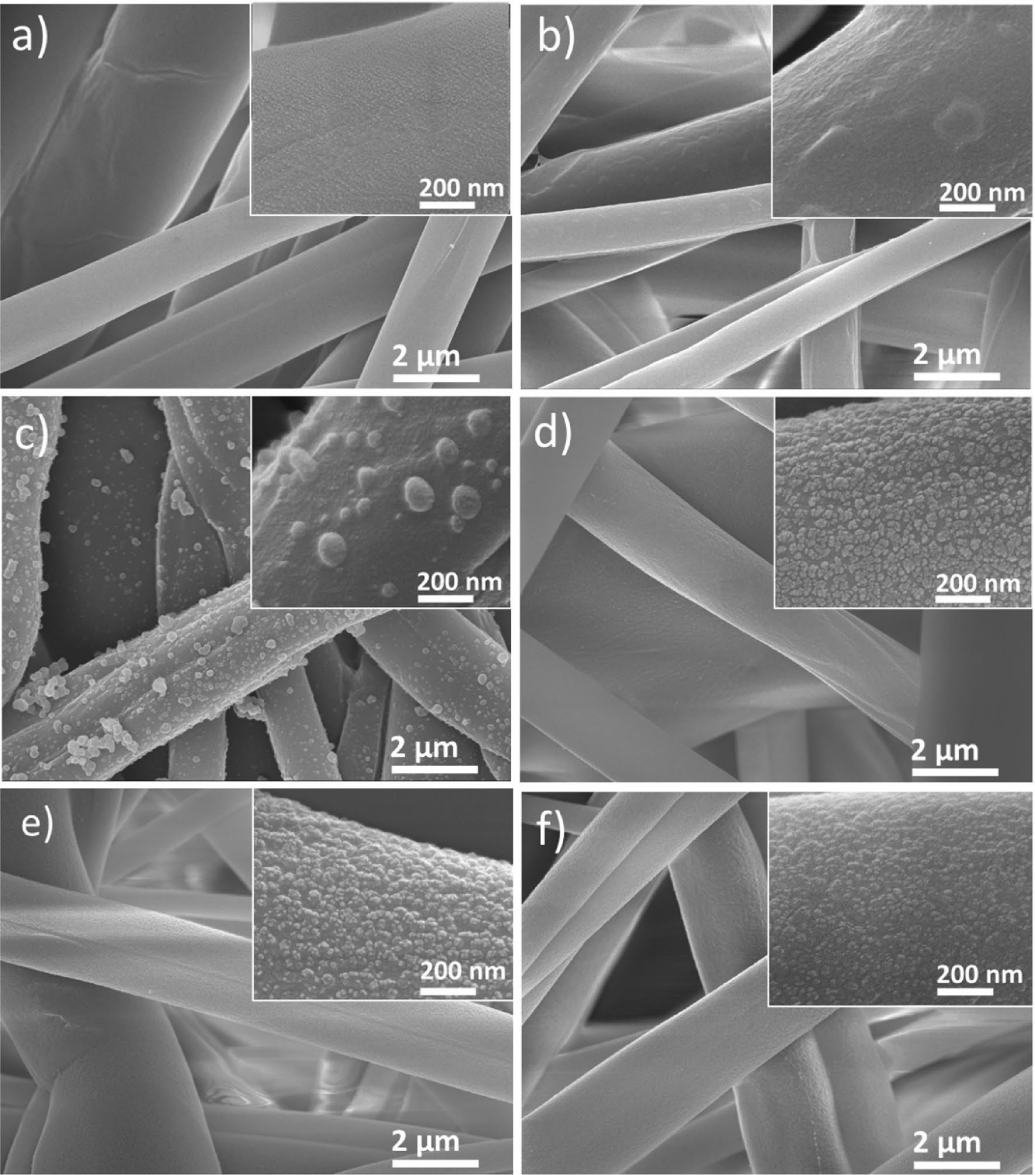
The SEM images for (**a**) PPMM, (**b**) PPMM@PVA, and (**c**) PPMM@PDA, as well as (**d**) PPMM@PDA/PVA(1/2), (**e**) PPMM@PDA/PVA(1/4), and (**f**) PPMM@PDA/PVA(1/8), respectively. The insets are their respective high magnification images.

Figure 1 displays the SEM images for PPMM, PPMM@PVA, PPMM@PDA, and PPMM@PDA/PVA(1/2, 1/4, 1/8), respectively. In Fig. 1a, the PPMM demonstrated a non-woven microstructure in which individual PP fibers revealed a smooth surface with fiber diameters between 0.9 and 6.5 μm. In Fig. 1b, the PPMM@PVA demonstrated a similar morphology as the deposition of PVA occurred exclusively on the external surface of PPMM. This was anticipated due to the hydrophilic nature of PVA that limited its infiltration and formation of intimate bonding with the hydrophobic PPMM. For PPMM@PDA, shown in Fig. 1c, there appeared many agglomerates in sizes of 60 ~ 300 nm on the surface of PP fibers. These agglomerates were PDA deposited from the solution via the self-polymerization of DA. Since the DA contained a phenol group, the PDA was likely to form CH_2_-π bonding with the PP fibers, resulting in a more pronounced deposition. However, the formation of PDA from DA involved an oxidation reaction from the participation of dissolved oxygen. As a result, the PDA formation was also observed on the external surface of PPMM. In Fig. 1d–f, the images of PPMM@PDA/PVA(1/2, 1/4, 1/8) demonstrated significantly improved surface morphologies, and the PDA agglomerates were formed uniformly on individual PP fibers. Since there was strong hydrogen bonding between the PVA and DA, the PVA was able to facilitate homogeneous formation of PDA with smaller agglomerate sizes. This allowed the PDA/PVA to infiltrate to the internal space of entire PPMM and produced a conformal coating on every PP fibers. Interestingly, with a smaller PDA/PVA ratio, the surface morphology became more refined with smaller PDA agglomerates. In our experiments, the smallest PDA/PVA mass ratio was 1/8 as additional PVA amounts caused preferential PDA deposition on the external surface of PPMM again.

**Figure 2:**
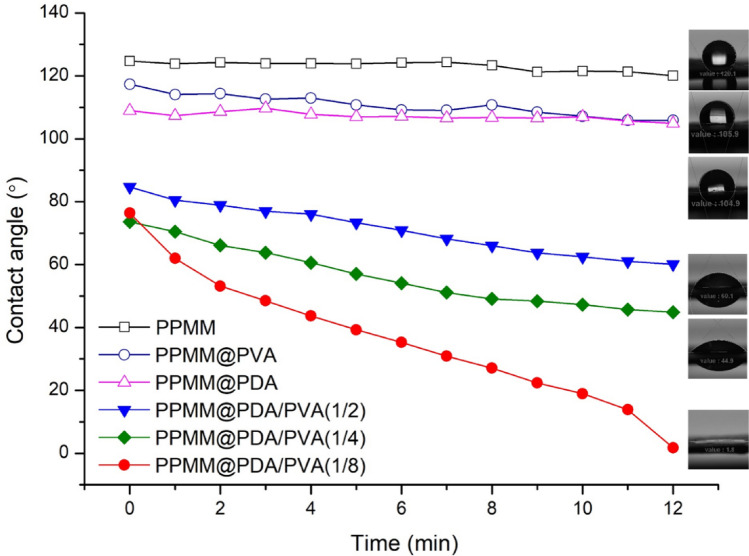
The variation of water contact angles as a function of time for PPMM, PPMM@PVA, and PPMM@PDA, as well as PPMM@PDA/PVA(1/2, 1/4, 1/8). The photographs are their respective water droplets taken at 12 min.

The hydrophilicity of functionalized PPMM is critical for successful deposition of Au and IrO_2_. Figure 2 displays the dynamic water contact angles for PPMM, PPMM@PDA, PPMM@PVA, and PPMM@PDA/PVA(1/2, 1/4, 1/8), respectively. The insets are the photographs for water droplets at 12 min. As shown, the PPMM exhibited a superhydrophobic behavior with a contact angle greater than 120°. This is anticipated as the PPMM consists of nonpolar PP fibers, and is designed for filtering of nonaqueous solution. After coating with PVA, the contact angle for PPMM@PVA was 117.4° initially, and became slightly reduced to 105.9° after 12 min. The hydrophobic behavior of PPMM@PVA suggested that the PVA was not forming a conformal coating on individual PP fibers of PPMM. Instead, only the PP fibers on the external surface of PPMM were partially covered, as evidenced by Fig. 1b. For PPMM@PDA, a similar pattern was recorded with an initial contact angle of 109° that decreased to 105° after 12 min. Again, the hydrophilic PDA agglomerates were predominately deposited on the external surface of PPMM, as evidenced by Fig. 1c, so the PPMM@PDA still revealed a hydrophobic behavior. In contrast, the PPMM@PDA/PVA(1/2, 1/4, 1/8) exhibited substantial improvements in hydrophilicity. For example, their starting contact angles were consistently smaller than 90°, and became even smaller as time progressed, suggesting a steady infiltration of water. Notably, with a smaller PDA/PVA ratio, the hydrophilicity became even more pronounced. For instance, the PPMM@PDA/PVA(1/8) revealed a notable hydrophilic behavior, and the water droplet became completely flat after 12 min. We recognized that the hydrophilic nature of PPMM@PDA/PVA(1/2, 1/4, 1/8) was associated with the conformal coating of PDA/PVA on the PP fibers. In particular, the PPMM@PDA/PVA(1/8) demonstrated the most desirable hydrophilic behavior, and therefore was selected as the flexible substrate for following deposition of Au and IrO_2_ nanoparticles.

**Figure 3:**
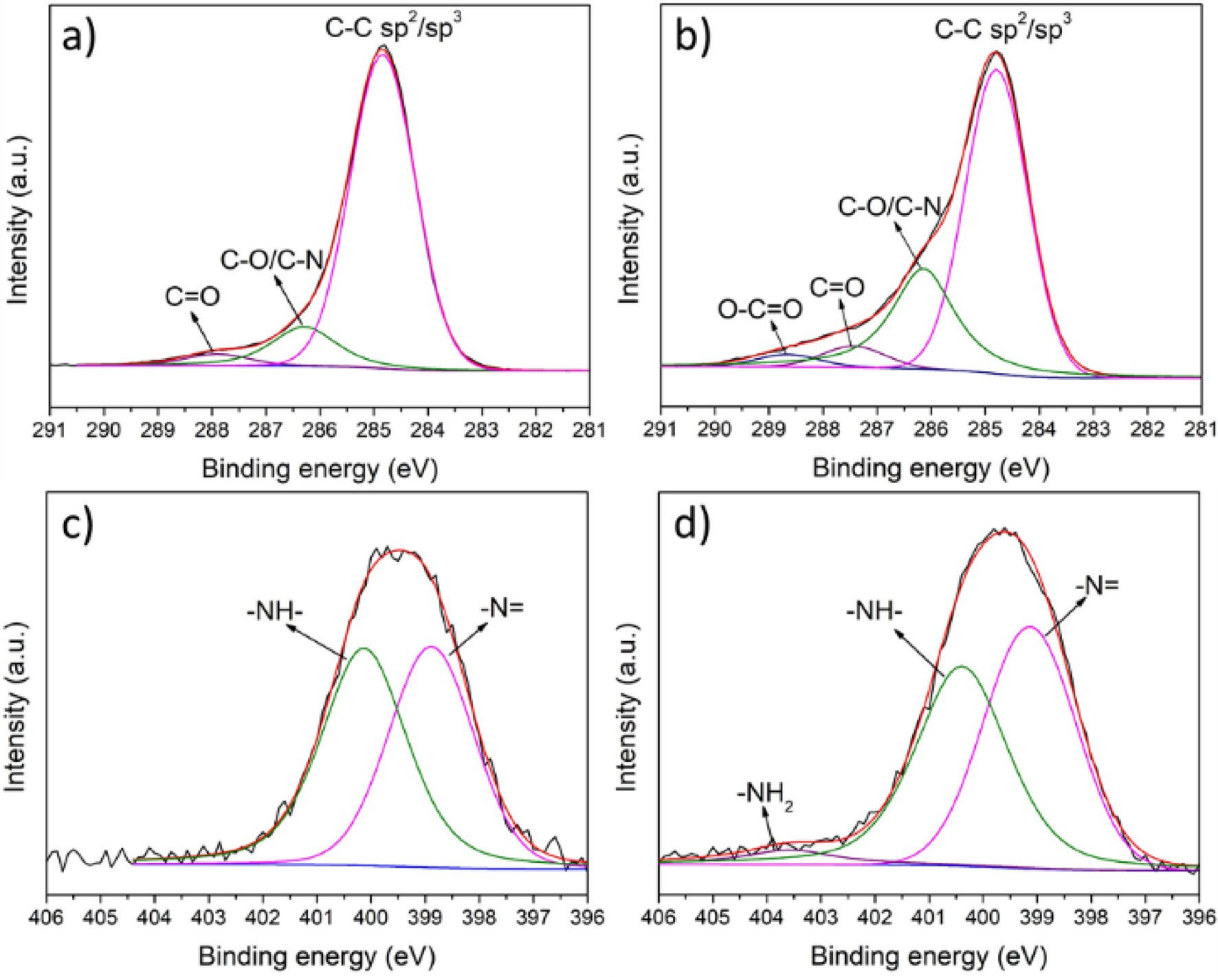
The XPS C1s profiles and fitting curves for (**a**) PPMM and (**b**) PPMM@PDA/PVA(1/8), as well as the XPS N1s profiles and fitting curves for (**c**) PPMM and (**d**) PPMM@PDA/PVA(1/8).

The XPS was employed to elucidate the chemical nature of functionalized PPMM. Figure 3 displays the XPS profiles and fitting curves for C1s and N1s from PPMM and PPMM@PDA/PVA(1/8). As shown in Fig. 3a, the C1s signal from PPMM revealed the characteristic C–C bonding from the primary molecular structure of PP. However, the curve fitting result indicated the presence of minor signals which were associated with the alcohol/amine (C–O/C–N) and ketone (C=O) bonds. Those peaks were presumably caused by the additives in the PPMM fabrication process.

In contrast, the curve fitting of C1s profile from PPMM@PDA/PVA(1/8), shown in Fig. 3b, exhibited stronger intensities for both C–O/C–N and C = O bonds, as well as moderate presence of carboxyl (O–C = O) bond. Since the PDA contains phenol, amine, and ketone groups in its chemical structure, and the PVA is synthesized by partial or fully hydrolysis of polyvinyl acetate, the coexistence of O–C = O, C–O, C = O, and C–N was reasonably expected^23,24^. Figure 3c,d display the N1s profiles for PPMM and PPMM@PDA/PVA(1/8), respectively. The curve fitting for PPMM, shown in Fig. 3c, exhibited two distinct peaks associated with –N = and –NH–, which were additives in the PPMM fabrication process. In Fig. 3d of PPMM@PDA/PVA(1/8), the predominant signal consisted of –N= and –NH–, but a minor one associated with –NH_2_ was also recorded. According to Liebscher et al., three distinct amine groups (primary, secondary, tertiary) might be present in PDA, contingent on its degree of oxidation^25^. Therefore, the presence of amine (–NH_2_) group and an increasing ratio of N/C provided the solid evidence validating the existence of PDA. Table 1 lists the XPS fitting results of C1s and N1s signals from PPMM and PPMM@PDA/PVA(1/8), respectively.

**Table 1:** The XPS fitting results of C1s and N1s profiles from PPMM and PPMM@PDA/PVA(1/8), respectively.

	Compound type			
	C–C sp^2^/sp^3^	C–O/C–N	C=O	O–C=O
PPMM	83.5 at%	13.1 at%	3.4 at%	0
PPMM@PDA/PVA(1/8)	63.8 at%	29.1 at%	4.5 at%	2.6 at%

	Compound type			Atomic ratio
	–N=	–NH–	–NH_2_	N/C
PPMM	47.2 at%	52.8 at%	0	0.057
PPMM@PDA/PVA(1/8)	48.3 at%	47.4 at%	4.3 at%	0.095

**Figure 4:**
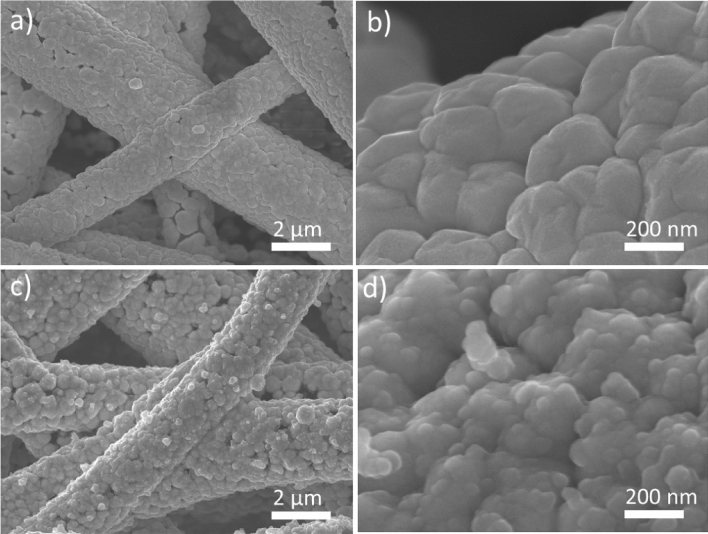
The SEM images for PPMM@PDA/PVA(1/8)@Au in (**a**) low and (**b**) high magnification views, as well as PPMM@PDA/PVA(1/8)@Au@IrO_2_ in (**c**) low and (**d**) high magnification views.

Figure 4 displays the SEM images for PPMM@PDA/PVA(1/8)@Au and PPMM@PDA/PVA(1/8)@Au@IrO_2_ in low and high magnification views. As shown in Fig. 4a, the PPMM@PDA/PVA(1/8)@Au in low magnification view demonstrated an uniform coating of Au. In high magnification view, shown in Fig. 4b, there appeared a notable coalescence of Au grains on individual PP fibers. The thickness of conformal Au overcoat was around 150 nm. In Fig. 4c, the PPMM@PDA/PVA(1/8)@Au@IrO_2_ in low magnification view exhibited a rough surface, a phenomenon attributed to the deposition of IrO_2_ nanoparticles. The high magnification view, shown in Fig. 4d, confirmed the presence of IrO_2_ nanoparticles on the surface of Au grains. In addition, the size of IrO_2_ nanoparticles was around 50 nm.

### Analysis of pH-sensing performance

**Figure 5:**
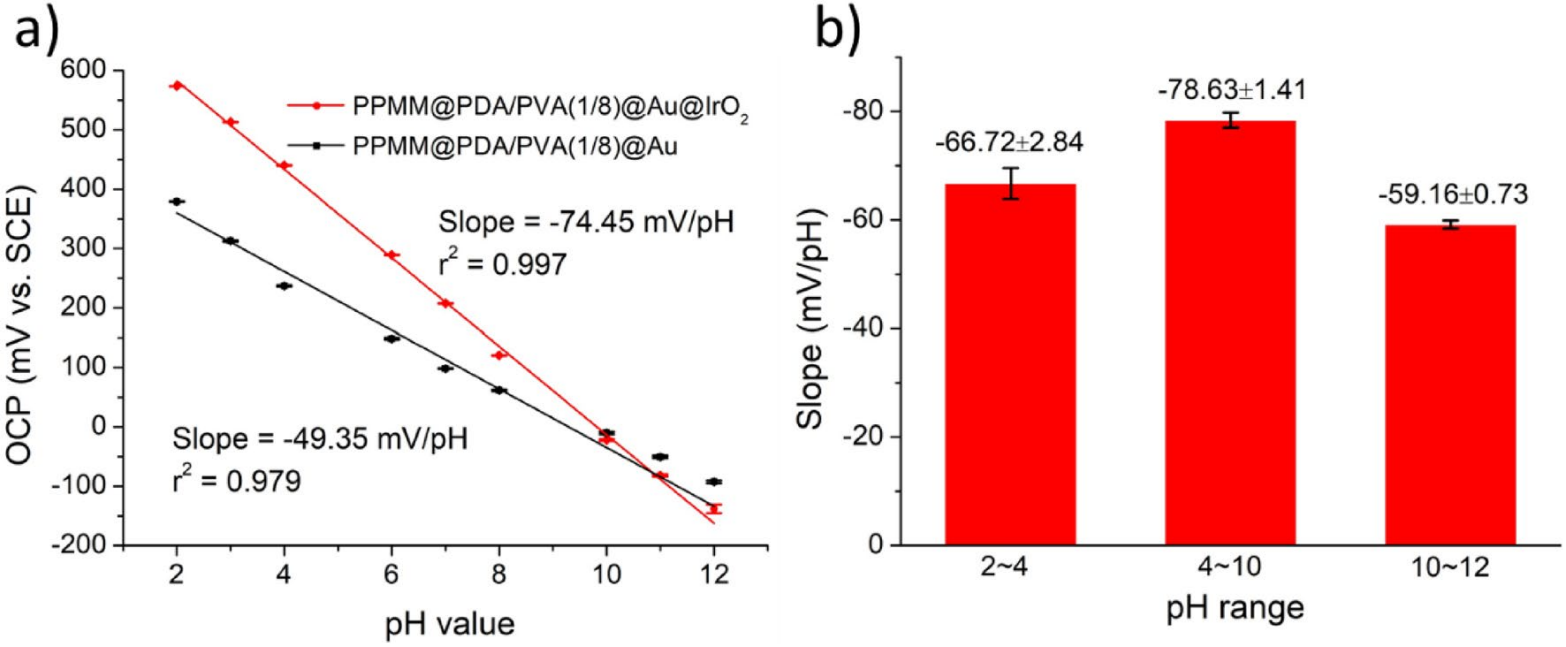
(**a**) The potentiometric response of PPMM@PDA/PVA(1/8)@Au and PPMM@PDA/PVA(1/8)@Au@IrO_2_ in aqueous solutions with pH values adjusted from 2 to 12. (**b**) The corresponding pH-sensing sensitivities of PPMM@PDA/PVA(1/8)@Au@IrO_2_ in aqueous solutions of different pH ranges.

Figure 5a displays the OCP of PPMM@PDA/PVA(1/8)@Au and PPMM@PDA/PVA(1/8)@Au@IrO_2_ in Britton-Robinson buffer solutions with pH values adjusted from 2 to 12. The corresponding pH-sensing slopes for both samples were − 49.35 and − 74.45 mV/pH, respectively. For pH-sensing, the Au was primarily used as a current collector instead of functioning as an active pH-sensing element. In addition, the Au was reported to reveal a pH sensing slope of − 53.4 mV/pH, a value that was rather close to what we observed^26^. On the other hand, the IrO_2_ has been established as an impressive pH-sensing material as it is known to show a super-Nernstian response. In the literature, the pH sensitivity of IrO_2_ was found to be affected by the preparation methods involved^27,28^. For example, from solution-based processes, the surface of IrO_2_ was mostly occupied by hydrate IrO_2_, and therefore a super-Nernstian behavior with a pH-sensing slope between − 60 and − 80 mV/pH was anticipated^29,30^. The redox potential behavior for super-Nernstian response is listed below.

2 $$E={E}_{0}-\frac{\mathrm{mRT}}{nF}pH$$

where the *E* is the redox potential, the *E*_*0*_ is the standard redox potential, the *n* and *m* are the numbers of electrons and protons involved in the redox reaction, the *R* is the ideal gas constant, the *T* is the absolute temperature, and the *F* is the Faraday constant. In the case of hydrated IrO_2_, the redox reaction is listed below^31^.

3 $${\mathrm{Ir}}_{2}\mathrm{O}{(\mathrm{OH})}_{3}{\mathrm{O}}_{3}^{3-}+3{\mathrm{H}}^{+}+2{\mathrm{e}}^{-}\leftrightarrow 2\mathrm{Ir}{(\mathrm{OH})}_{2}{\mathrm{O}}^{-}+{\mathrm{H}}_{2}\mathrm{O}$$

We substituted those known constants into the equation, and the theoretical pH-sensing slope could be expressed as;

4 $$\left(-\mathrm{m}/\mathrm{n}\right)\left(\frac{RT}{F}\right)=\left(-3/2\right)(0.05916)=-88.7\frac{mV}{pH}, \quad at \,\, 25\,^\circ{\rm C}$$

In our pH-sensing results, the PPMM@PDA/PVA(1/8)@Au@IrO_2_ exhibited a super-Nernstian response with a pH-sensing slope of − 74.45 mV/pH. This value was slightly smaller than the theoretical value of − 88.7 mV/pH because according to our earlier XPS analysis, our sample was consisted of both hydrated and anhydrous IrO_2_^32^. It is known that the anhydrous IrO_2_ reveals an inferior sensing response to that of hydrated IrO_2_^27^. The redox reaction of anhydrous IrO_2_ is listed in Eq. (5), and the sensing slope is expressed in Eq. (6) as − 59.1 mV/pH. Therefore, the expected overall sensitivity for our sample is between − 59.1 and − 88.65 mV/pH.

5 $${2\mathrm{IrO}}_{2}+2{\mathrm{H}}^{+}+2{\mathrm{e}}^{-}\leftrightarrow {{\mathrm{Ir}}_{2}\mathrm{O}}_{3}+{\mathrm{H}}_{2}\mathrm{O}$$

6 $$\left(-\mathrm{m}/\mathrm{n}\right)\left(\frac{RT}{F}\right)=\left(-2/2\right)(0.05916)=-59.1\frac{mV}{pH}, \quad at \,\, 25^\circ{\rm C}$$

We also obtained the pH-sensing slopes in different pH ranges to further explore the pH sensitivity of PPMM@PDA/PVA(1/8)@Au@IrO_2_. Figure 5b displays the corresponding bar charts of pH-sensing slope in aqueous solutions with different pH values. Apparently, the PPMM@PDA/PVA(1/8)@Au@IrO_2_ revealed a higher slope of − 78.63 mV/pH in solutions with the pH range of 4 to 10. It is noted that the pH values for human’s blood and urine are 7.3 to 7.4 and 4.5 to 7.8, respectively^33,34^. Thus, our PPMM@PDA/PVA(1/8)@Au@IrO_2_ not only is suitable for wearable electronics but also is expected to be potentially useful as a pH sensor in in vivo environment.

**Figure 6:**
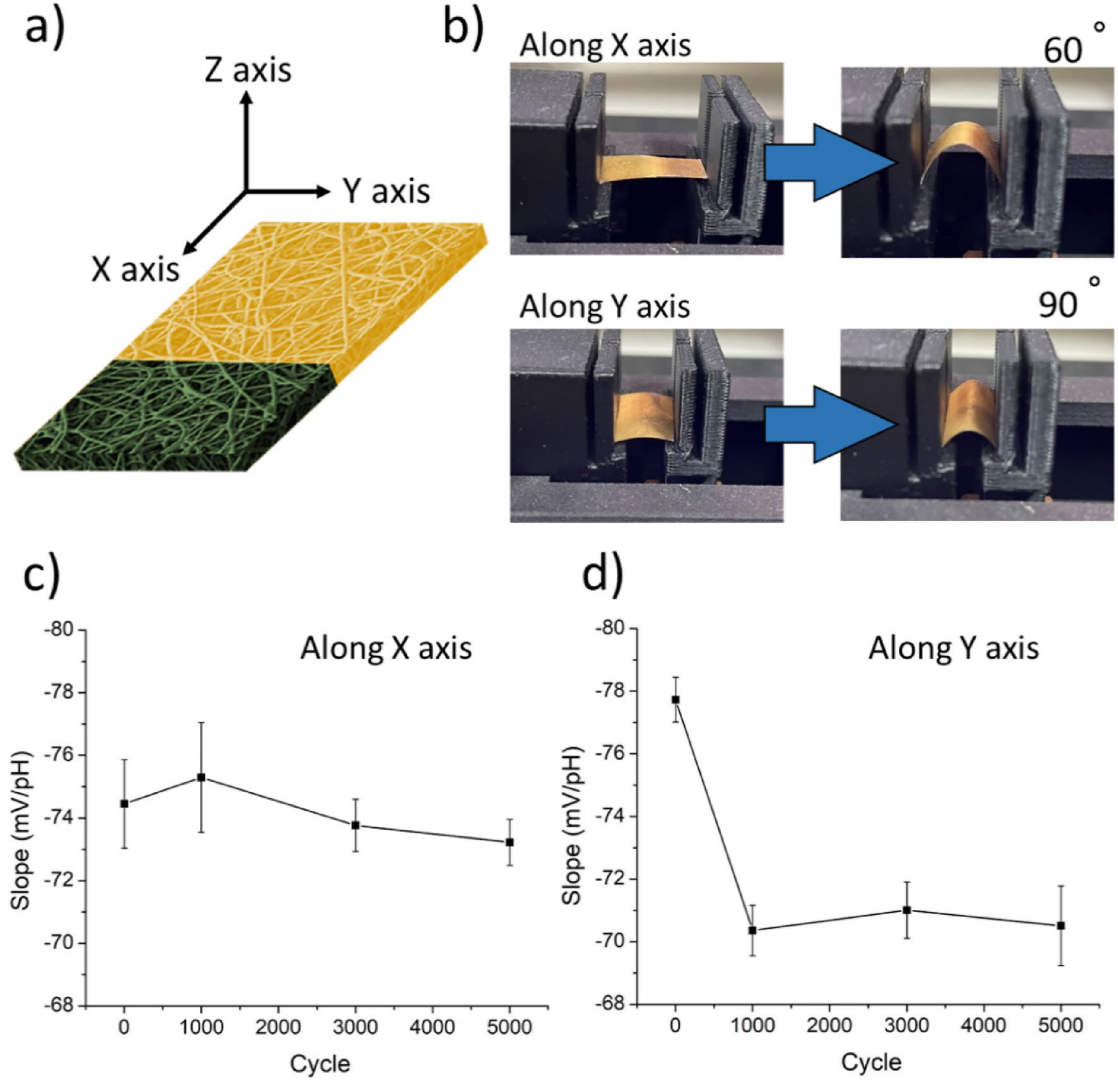
(**a**) The PPMM@PDA/PVA(1/8)@Au@IrO_2_ with two bending directions (X axis and Y axis). The green color represents the area where IrO_2_ nanoparticles are actually deposited. (**b**) The photographs for bending tests along the X axis and Y axis, respectively. The resulting pH-sensing slope and standard deviation as a function of bending cycles along the (**c**) X axis and (**d**) Y axis.

To validate the flexibility of PPMM@PDA/PVA(1/8)@Au@IrO_2_ and its pH-sensing performance after multiple bending cycles, we tested the sample under two different bending directions, as shown in Fig. 6a. Figure 6b displays the photographs for bending tests in which the sample was bended along the X axis and Y axis to 60° and 90°, respectively. We recorded the pH-sensing slopes at the 0th, 1000th, 3000th, and 5000th cycles, respectively. Figure 6c displays the corresponding pH-sensing slopes and standard deviations for PPMM@PDA/PVA(1/8)@Au@IrO_2_ undergoing bending action along the X axis. The sensitivity over repeated bending was relatively steady without noticeable deterioration, an expected behavior as any physical deformation was confined on the metalized PPMM exclusively. In contrast, for the sample undergoing bending along the Y axis, the area undergoing deformation covered both metalized PPMM and IrO_2_. The corresponding pH-sensing behavior, as shown in Fig. 6d, revealed minor deterioration at the 1000th cycle, followed by stable sensing responses afterwards. Nonetheless, the pH-sensing slope still retained the desirable super-Nernstian response within 5000 bending cycles, which validated the flexible and durable nature of PPMM@PDA/PVA(1/8)@Au@IrO_2_.

**Figure 7:**
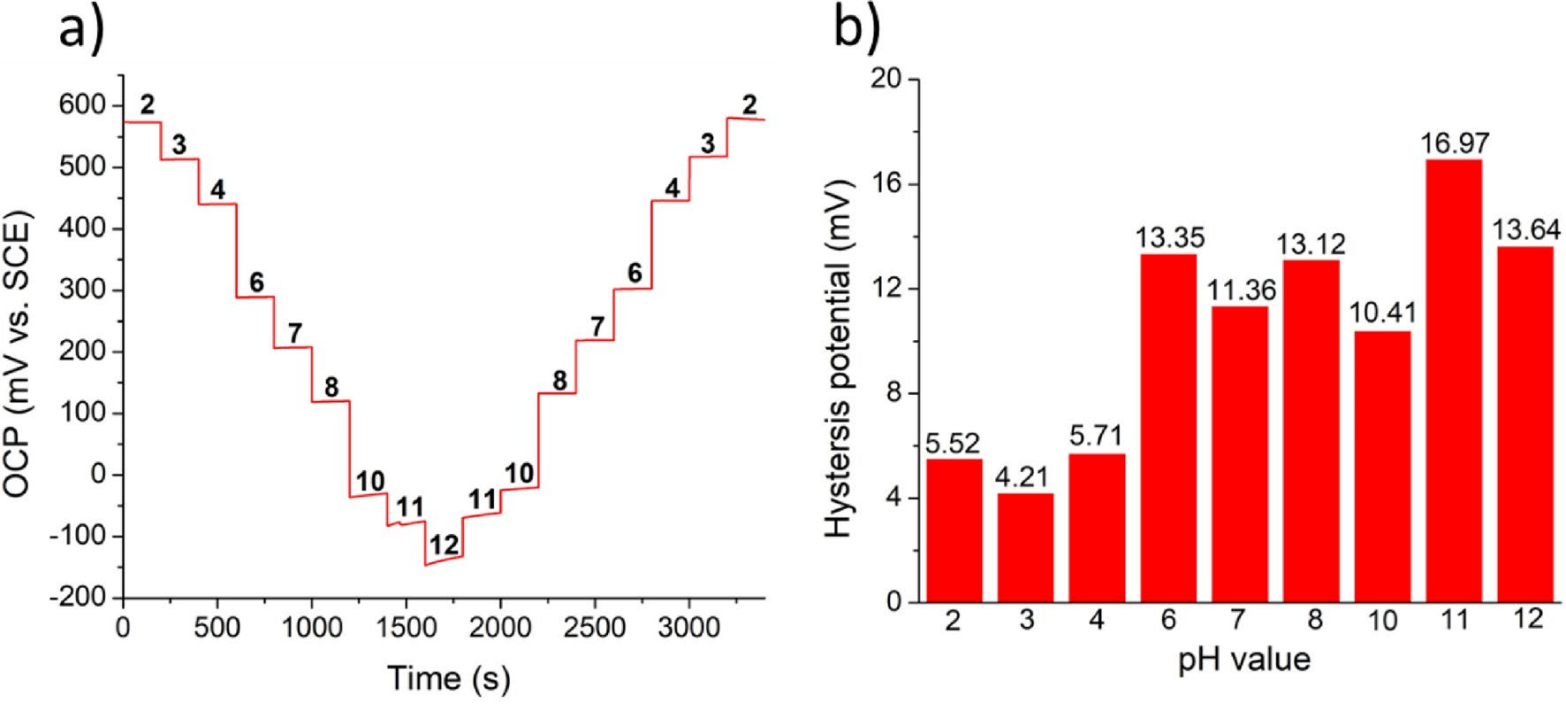
(**a**) The testing cycle for PPMM@PDA/PVA(1/8)@Au@IrO_2_ in Britton-Robinson buffer solutions of pH 2–3–4–6–7–8–10–11–12–11–10–8–7–6-4–3–2. (**b**) The hysteresis potential (dV) at the respective pH level.

Figure 7a displays the OCP of PPMM@PDA/PVA(1/8)@Au@IrO_2_ in Britton-Robinson buffer solutions with different pH values. The sample was immersed in the solution in which the pH value was adjusted in the order of pH 2–3-4–6-7–8-10–11-12–11-10–8-7–6-4–3-2. The hysteresis behavior, defined as the hysteresis potential (dV), is determined by the difference of the OCPs when the sample is immersed at different times in the solution with identical pH value^35^. Figure 7b displays the hysteresis potentials at respective pH values. Apparently, under an acidic solution, the sample revealed a relatively smaller hysteresis potential, which was likely caused by the faster ionic exchange of protons in an acidic solution^36^. In a neutral solution, the concentration of proton or hydroxyl ion was relatively low which led to a slower ionic exchange rate on the electrode surface. The greater hysteresis phenomena in an alkaline solution was attributed to the strong interaction between the hydroxyl groups and oxide surface as the IrO_2_ was unstable in an alkaline solution^37^.

**Figure 8:**
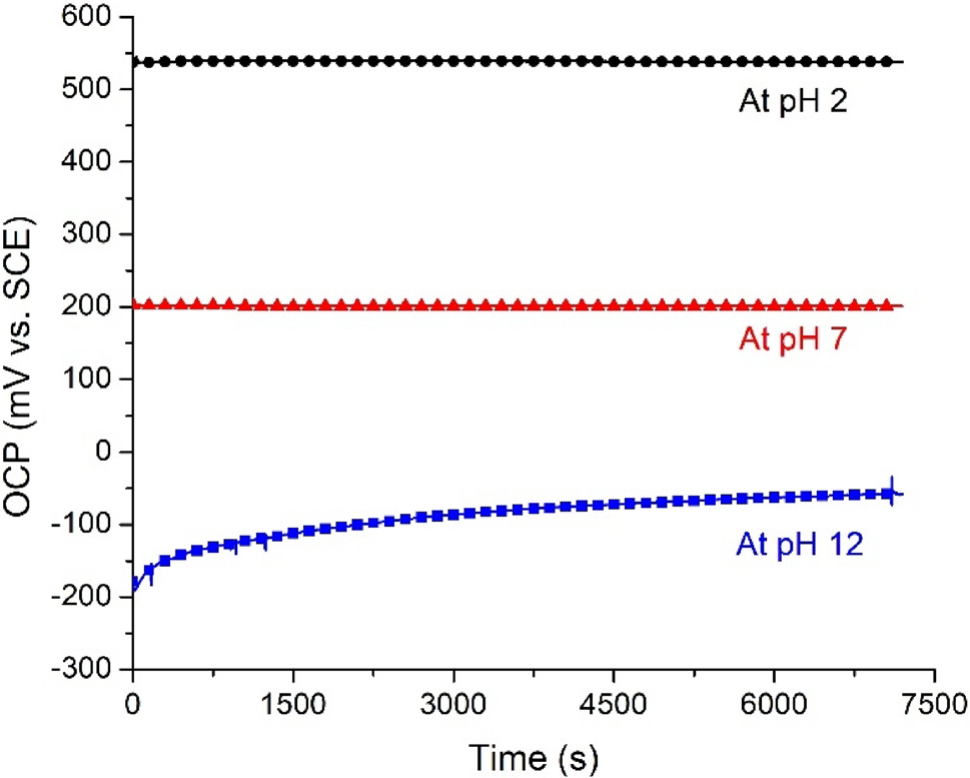
Drift effect of PPMM@PDA/PVA(1/8)@Au@IrO_2_ in aqueous solutions at pH values of 2, 7, and 12 for 7200 s.

To investigate the stability of our sample, we measured the variation of OCP in acidic, neutral, and alkaline solutions. Figure 8 displays the drift effect at pH values of 2, 7, and 12 for 7200 s. The drift potential was obtained by comparing the average potential for the final 200 s and the first 200 s. The drift rate at pH values of 2, 7, and 12 was 0.14, −0.76, and 57.75 mV/h, respectively. Apparently, our sample revealed an impressive stability in both acidic and neutral solutions. However, at pH 12, the hydrated IrO_2_ became unstable so the drift behavior was rather poor. Since in the in-vivo condition, the body fluid is either neutral or weakly acidic, the drift performance for our sample is proven to be useful for implantable electronics.

We have compared our sample with what have been published in the literature. The comparison is listed in Table 2 below. As listed, among these flexible pH sensors, our sample demonstrates a superior sensitivity which is attributed to the presence of hydrated IrO_2_ from our wet chemical synthetic route. It is known that hydrated IrO_2_ exhibits a better pH sensing behavior than that of anhydrous IrO_2_.

**Table 2:** The comparison of pH sensing sensitivity among different flexible pH sensors in the literature.

	pH sensitivity (mV/pH)	pH sensing range	References
Our sample	− 74.45	2–12	This work
Flexible PANI membrane	− 58.57	5.45–8.62	^38^
Flexible IrO_x_/AgCl electrode	− 51.7	1.5–12.1	^28^
Polyaniline nanopillar sensor	− 60.3	2.09–12.0	^39^
Flexible graphene pH sensor	− 62	5–8	^40^
Au composite strip-based sensor	− 57	3–8	^41^

**Figure 9:**
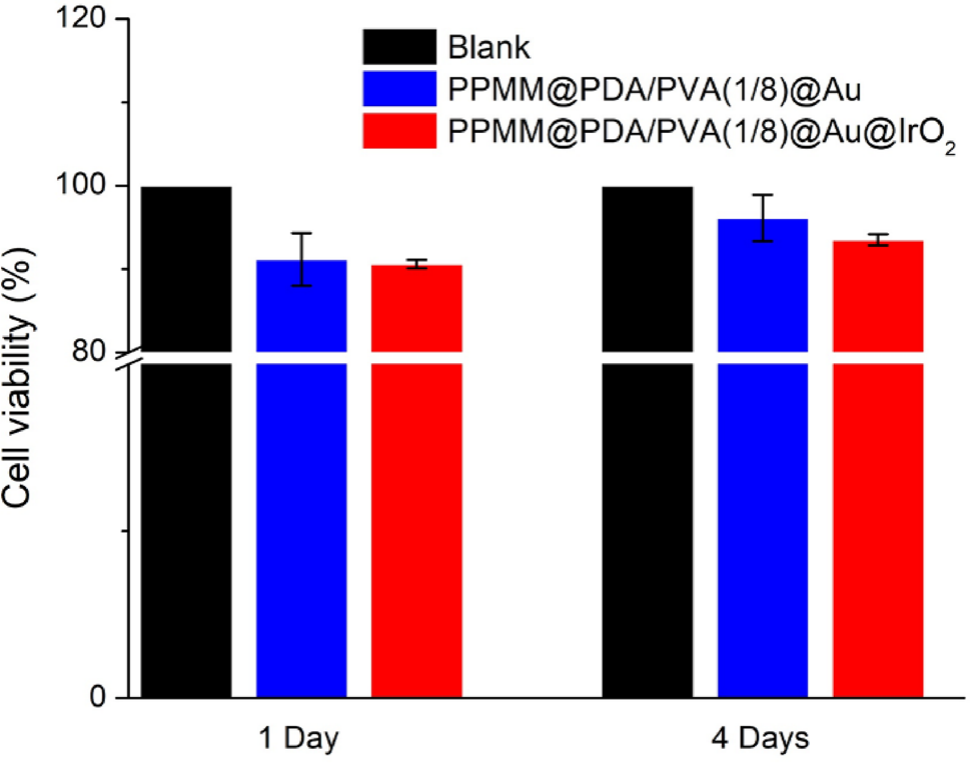
The bar chart of cell viability for the control group (blank), PMM@PDA/PVA(1/8)@Au, and PPMM@PDA/PVA(1/8)@Au@IrO_2_.

Figure 9 displays the bar chart for cell viability experiments. After 1 day of cell culture, the cell viability for PPMM@PDA/PVA(1/8)@Au and PPMM@PDA/PVA(1/8)@Au@IrO_2_ was 91.2% and 90.6% of that of blank sample, which served as the control group. Interestingly, after 4 days, their cell viability was slightly increased to 96.1% and 93.5%, respectively. This moderate improvement in cell viability was possibly attributed to the presence of IrO_2_ because the IrO_2_ was reported to promote cell growth in the literature^42^. From our results, both PPMM@PDA/PVA(1/8)@Au and PPMM@PDA/PVA(1/8)@Au@IrO_2_ revealed impressive biocompatibility, which was defined with a cell viability above 80%.

In fabricating the flexible pH sensor for possible uses in wearable and implantable electronics, the materials we employed (PPMM, PDA, PVA, Au, IrO_2_) are known for their biocompatibility. In addition, the chemicals involved during the synthetic process were deliberately chosen to minimize any residual toxicity. For example, a typical electroless Au deposition requires a sequential sensitization and activation process using Sn and Pd ions to prepare Pd nuclei for subsequent Ni electroless deposition^43^. The Ni was then served as the seeds for Au deposition. However, both Pd and Ni exhibit moderate biotoxicity so their presence is not desirable. Therefore, the use of PDA for Au nuclei formation gave us an alternative biocompatible route without involving toxic chemicals for sensitization and activation. Since the PDA was deposited on the surface of PP fibers, the reduction of Au^3+^ ions for Au nuclei took place exclusively on the PP fibers instead of homogeneously in the solution. In addition, conventional electroless Au depositions entail reducing agents such as N_2_H_4_ and NH_2_OH·HCl, and those chemicals are not biocompatible. On the contrary, in our process the reducing agent was the sodium L-ascorbate because it not only reveals a mild reducing power but also is known for its biocompatibility.

It is further noted that during pH sensing, the IrO_2_ is not susceptible to interference from monovalent cations (Na^+^, K^+^) and divalent cations (Mg^2+^)^28,44,45^. In addition, the selection of PPMM over conventional polymeric substrates such as polyimide (PI), polyamide (PA), polytetrafluoroethylene (PTFE), polyethylene terephthalate (PET), and polyvinylchloride (PVC) is based on its relatively low cost, impressive corrosion resistance, and excellent biocompatibility. More importantly, the porous structure of PPMM could be readily tailored to meet specific applications.

## Conclusions

A flexible pH sensor was fabricated by carrying out deposition of Au and IrO_2_ on individual PP fibers of functionalized PPMM. Both PDA and PVA were used as the surface agents to improve the hydrophilicity of otherwise hydrophobic PPMM. The SEM images validated the formation of continuous Au overcoat on individual PP fibers, and the deposition of IrO_2_ nanoparticles with moderate coalescence. Contact angle measurements indicated that a molar ratio of PDA/PVA = 1/8 rendered the most hydrophilic PPMM. The pH-sensing behaviors were explored in aqueous solutions with the pH values adjusted between 2 and 12. The PPMM@PDA/PVA(1/8)@Au@IrO_2_ revealed a super-Nernstian response for a sensing slope of − 74.45 mV/pH. In addition, the PPMM@PDA/PVA(1/8)@Au@IrO_2_ maintained its sensing ability after repeated bending up to 5000 cycles. The PPMM@PDA/PVA(1/8)@Au@IrO_2_ revealed a subdued hysteresis behavior in an acidic solution. The raw materials and chemicals used in our sample preparation were deliberately chosen for their compatibility and the resulting PPMM@PDA/PVA(1/8)@Au@IrO_2_ was validated in cell viability test.
